# 156. Prevalence of Carbapenem Resistance Among The Clinical Isolates From Patients With Cardio Vascular Diseases Using X-pert Carba -R Real Time Multiplex PCR Assay in a Tertiary Care Hospital In Tamilnadu

**DOI:** 10.1093/ofid/ofad500.229

**Published:** 2023-11-27

**Authors:** Gerard J Rakesh, anusha rohit, Suresh Kumar Dorairajan, jaya prasanthi

**Affiliations:** Madras Medical Mission, Puducherry, Puducherry, India; The Madras Medical Mission, Chennai, Tamil Nadu, India; Apollo hospital, Chennai, Tamil Nadu, India; The Madras Medical Mission, Chennai, Tamil Nadu, India

## Abstract

**Background:**

In the Post Antibiotic era with the ongoing Silent pandemic of AMR, early detection of the resistant gene exhibited by the offending pathogen causing the ailment is of paramount importance. Early diagnosis and formulating the choice of antibiotics in treatment of Multi drug and Pan drug resistant bacteria helps in reducing the hospital stay and the overall outcome of the patients.

Carbapenems serve as the first line drugs for the treatment of drug resistant Gram-negative bacilli (GNB) infections.However, carbapenem resistance (CR) among GNB is quite common in India.CR can be due to carbapenemase production.The most common carbapenemases are

*bla*_KPC_, *bla*_NDM_, *bla*_VIM_, *bla*_IMP-1_, and *bla*_OXA-4._The present study aims to find the prevalence of Carbapenem resistance thereby aiding in initiating the empirical therapy. By studying the resistance patterns we would be able to know whether the isolate expresses single carbapenemase or multiple carbapenemases which has direct influence in the choice of antimicrobials and patient outcomes.

**Methods:**

A total of One hundred and seventy seven clinical isolates which were Carbapenem non-susceptible *Enterobacteriales, Pseudomonas aeruginosa, Acinetobater baumanii* and *Stenotrophomonas maltophilia* isolated during the period of Jan2022- April 2023 (15 months) were included in the study. Carbapenem resistance was confirmed by Vitek 2 automated AST system.These isolates were further subjected to X-pert Carba -R Real Time Multiplex PCR Assay to detect the type of carbapenamses expressed

**Results:**

Out of one hundred and seventy seven clinical isolates, one hundred and eight isolates did not produce carbapenemases by X-pert Carba -R Real Time Multiplex PCR Assay. A total of sixty nine isolates(38%) were positve for carbapenemases. Among the sixty nine isolates, thirty isolates(43%) produced *bla_NDM,_* fourteen isolates(20%) produced *bla_OXA-4_*.One isolate produced bla*_IMP-1_*.Twenty four isolates produced both *bla_NDM_* and *bla_OXA-4._*

**Conclusion:**

In Madras Medical Mission , New Delhi Metallo-Betalactamase -1 is the most common carbapenemase produced by the clinical isolates from Cardio-Vascular patients.Second most common carbapenemase is OXA-48.

**Disclosures:**

**All Authors**: No reported disclosures

